# Suprascapular nerve entrapment caused by a large hematoma of the scapula: a case report

**DOI:** 10.1186/s12891-023-06723-0

**Published:** 2023-07-19

**Authors:** Yong Bum Joo, Woo Yong Lee, Hyung Jin Chung

**Affiliations:** 1 Department of Orthopedic Surgery, Chungnam National University Hospital, Chungnam National University School of Medicine, Daejeon, South Korea; 2 Department of Orthopedic Surgery, Chungnam National University Sejong Hospital, Chungnam National University School of Medicine, Daejeon, South Korea

**Keywords:** Suprascapular nerve entrapment, Spingoglenoid notch, Hematoma, Open surgical decompression, Case report

## Abstract

**Background:**

Suprascapular nerve entrapment is a rare disorder that is frequently misdiagnosed as another disease. The suprascapular nerve is commonly entrapped at the following two sites: the suprascapular and spinoglenoid notches. Nerve entrapment at the spinoglenoid notch causes infraspinatus muscle weakness and atrophy. Patients present with posterior shoulder pain and weakness. Magnetic resonance imaging is used to confirm the diagnosis of a spinoglenoid cyst and nerve compression. Open or arthroscopic aspiration or decompression is indicated for patients with cysts in whom conservative treatment has failed and those with cysts associated with suprascapular nerve compression.

**Case presentation:**

Herein, we describe the case of a 49-year-old man with suprascapular nerve entrapment caused by a large cyst, namely, a hematoma, in the superior scapular and spinoglenoid notches. Open surgical decompression of the suprascapular nerve was performed owing to an intact rotator cuff and glenoid labrum.

**Conclusion:**

Posterior shoulder pain promptly resolved without complications.

## Background

The suprascapular nerve is a mixed motor and sensory nerve that originates from the brachial plexus (C5–6). The nerve crosses between the suprascapular notch and superior transverse scapular ligament into the posterior surface of the scapula, which dominates the supraspinatus and infraspinatus muscles [1]. Suprascapular nerve entrapment is a rare disorder that is frequently misdiagnosed as another disease [2]. Recently, cases of posterior shoulder discomfort and weakness, such as scapular dyskinesis, have caught the attention of orthopaedics. The suprascapular nerve is commonly entrapped in the following two sites: the suprascapular and spinoglenoid notches [2]. If the nerve is entrapped in the suprascapular notch, it induces supraspinatus and infraspinatus muscle weakness [3, 4]. In the case of entrapment in the spinoglenoid notch, infraspinatus muscle weakness alone is induced [4, 5]. Furthermore, suprascapular nerve compression is believed to be responsible for chronic shoulder pain [6].

Multiple etiologies of suprascapular nerve compression exist, with various mechanisms depending on the location of the compression. Ganglion cysts, direct nerve trauma, scapular fractures, vascular malformation, and iatrogenic injury potentially cause suprascapular nerve neuropathy at compression sites and the suprascapular and spinoglenoid notches [4, 7, 8].

Certain authors have reported that solitary paralabral cysts are a common cause of suprascapular nerve entrapment in relation to SLAP lesions [4, 8]. Conservative treatments, such as analgesics, manual therapy, and ultrasound-guided minimally invasive cyst aspiration combined with exercise, have demonstrated functional improvements [4, 7, 8]. However, surgical treatment can be considered if repeated conservative treatments prove ineffective. Traditionally, nerve release is performed under arthroscopy or with an open surgical technique [4, 8].

Herein, we present a unique case of a large cyst overlying both the suprascapular and spinoglenoid notches, causing infraspinatus atrophy in a patient who often played tennis. Open surgical decompression was performed. The patient’s pain was relieved and full recovery of shoulder function achieved.

## Case presentation

**Fig. 1:**
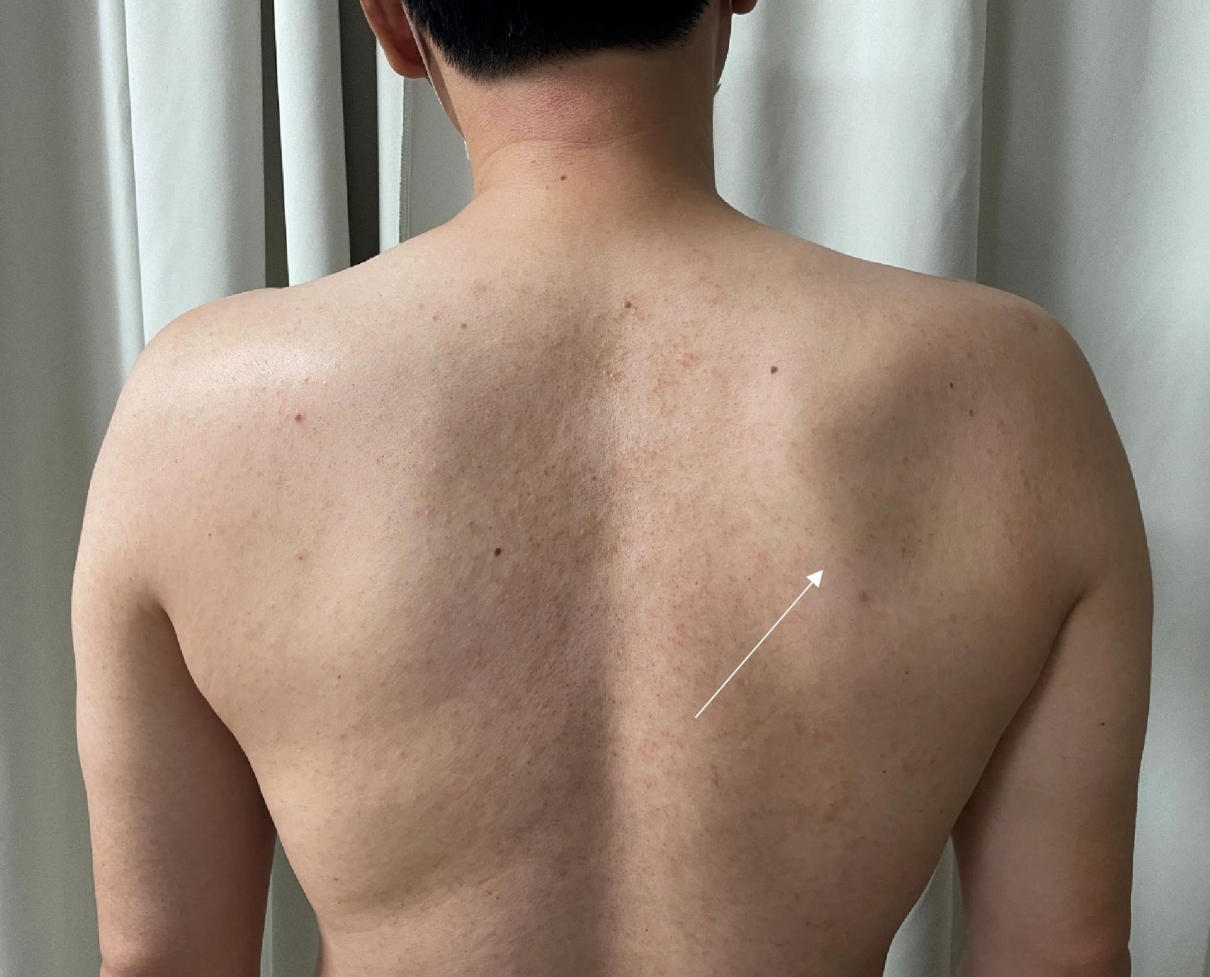
Clinical image of the patient’s right shoulder exhibiting infraspinatus muscle atrophy (line arrow)

**Fig. 2:**
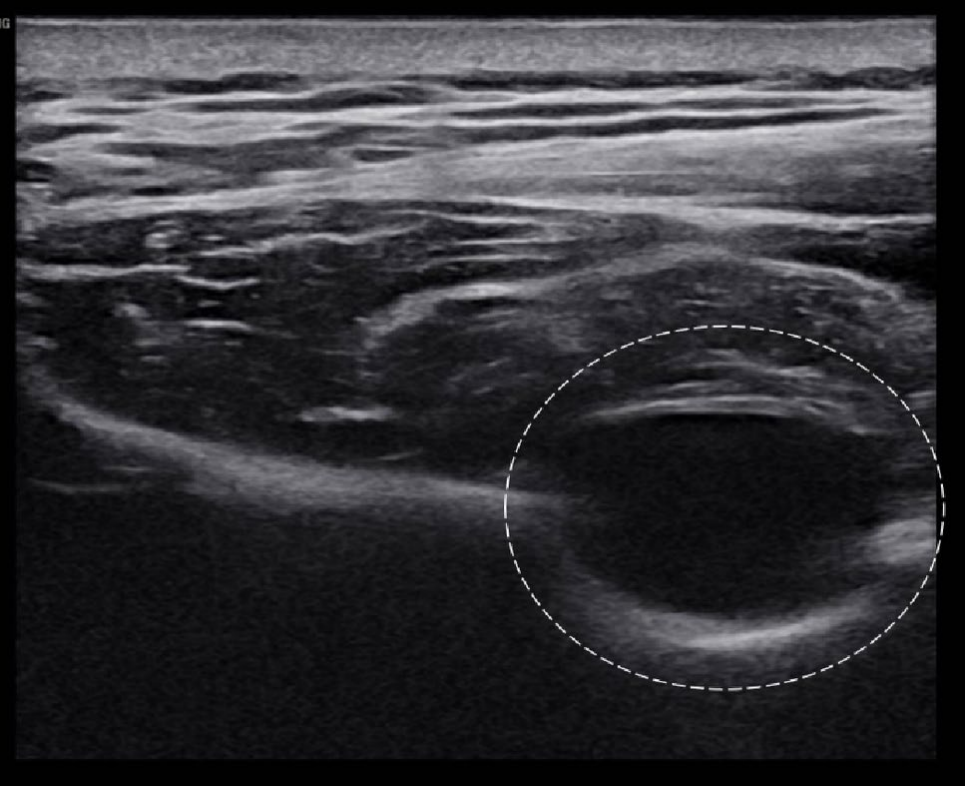
Ultrasound image showing a hypoechoic cyst near the suprascapular notch. (white circle)

A 49-year-old man presented with a 10-month history of discomfort in his right posterior shoulder. The patient also complained of discomfort while performing activities that required raising their right arm overhead. Initially, the patient would experience pain in their posterior shoulder after playing tennis (The patient was an office worker who had played tennis as a hobby for several years). On visiting our hospital, the patient could move their shoulder actively at 160° of flexion and abduction, 60° of external rotation at the side, and 90° of external rotation during abduction. On physical examination, the patient was able to maintain muscle strength around the shoulder. Also, there was no sensory impairment around the shoulder area. However, muscular atrophy of the infraspinatus muscle was observed over the scapular area (Fig. 1). In a previous ultrasound examination conducted at another orthopedic hospital that the patient had visited prior to visiting our hospital, a cystic lesion was detected near the scapular spine (Fig. 2). Ultrasound-guided aspiration was performed repeatedly, revealing bloody aspirated contents.

**Fig. 3:**
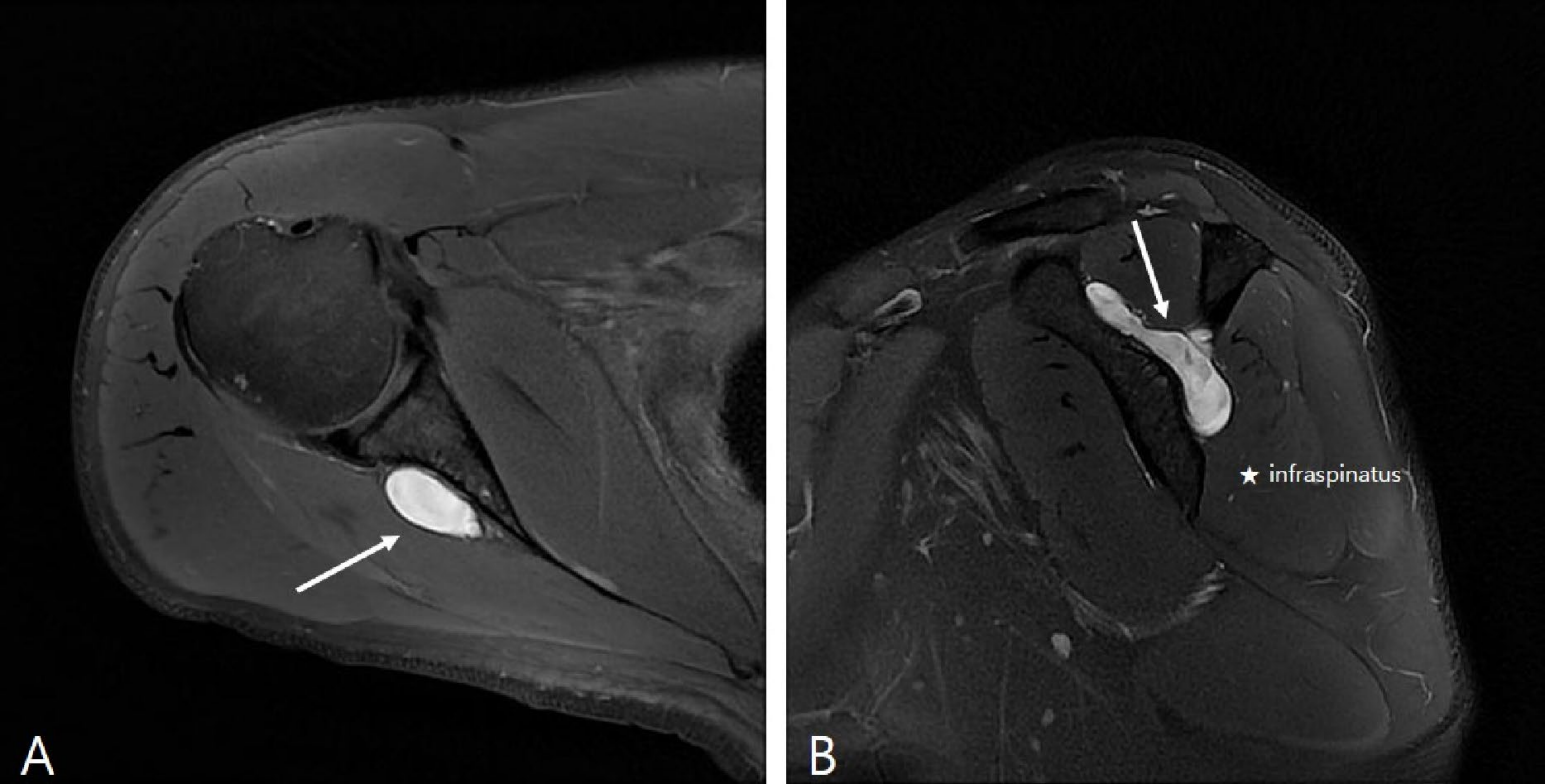
Preoperative magnetic resonance image of the lesion **A** The lesion (line arrow) exhibits high signal intensity on T2-weighted axial imaging. **B** Sagittal section revealing the heterogenous signal intensity of the cyst (line arrow) with the scapular spine of the right shoulder and infraspinatus muscle (star) denervation

Plain radiographs, including the true anterior–posterior, internal-rotation, and axillary views, revealed no abnormalities. Magnetic resonance imaging (MRI) revealed a huge loculated cystic lesion with high intensity on T2-weighted images and low intensity on T1-weighted images (3 × 2 × 4 cm). The cystic lesion was located over a wide range, from the suprascapular notch to the spinoglenoid notch (Fig. 3).

The infraspinatus muscles were atrophic with minimal fatty infiltration. None of the findings were consistent with glenoid labral tear or rotator cuff pathology. Electromyography and nerve conduction (EMG-NCV) examinations were performed with respect to muscle atrophy on visual examination; but there were no specific findings. (EMG showed no specific findings in the supraspinatus muscle and infraspinatus muscle, which was not compatible with damage to the suprascapular nerve. Also, the nerve conduction to the right arm was within normal limits.)

**Fig. 4:**
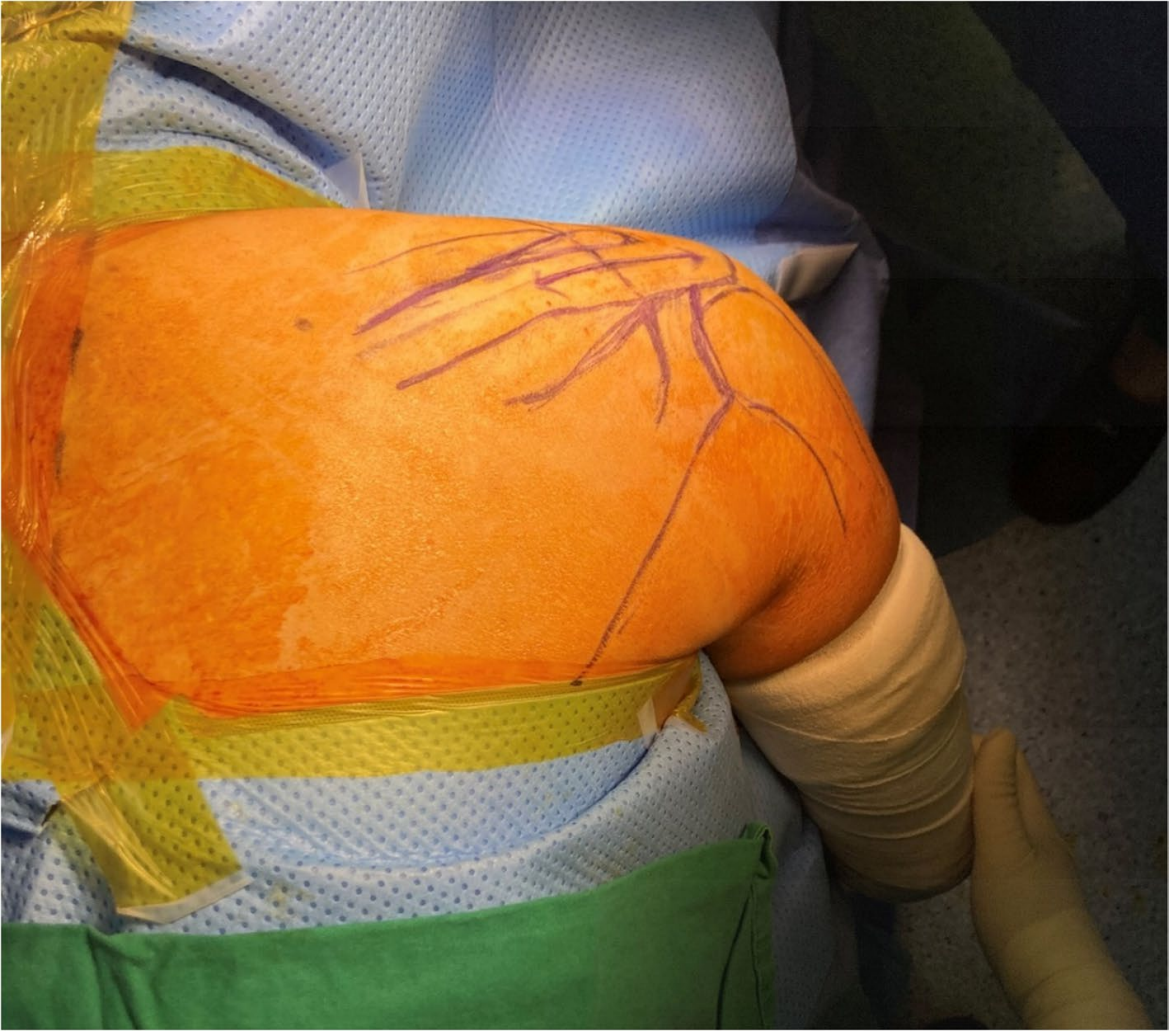
Patient in prone position with the incision made along the scapular spine line

On selecting the most appropriate treatment method, several factors were taken into consideration: 1) the large size of the cyst, that is, the hematoma; 2) its wide range of location; 3) the normal rotator cuff and labrum; and 4) clinical discomfort that persisted despite 10 months of nonsurgical treatment. Accordingly, open cyst decompression employing the posterior approach with the patient in the prone position was the optimal approach. Inasmuch as the cyst was located above and below the scapular spine, an incision was made along the scapular spine line (Fig. 4).

**Fig. 5:**
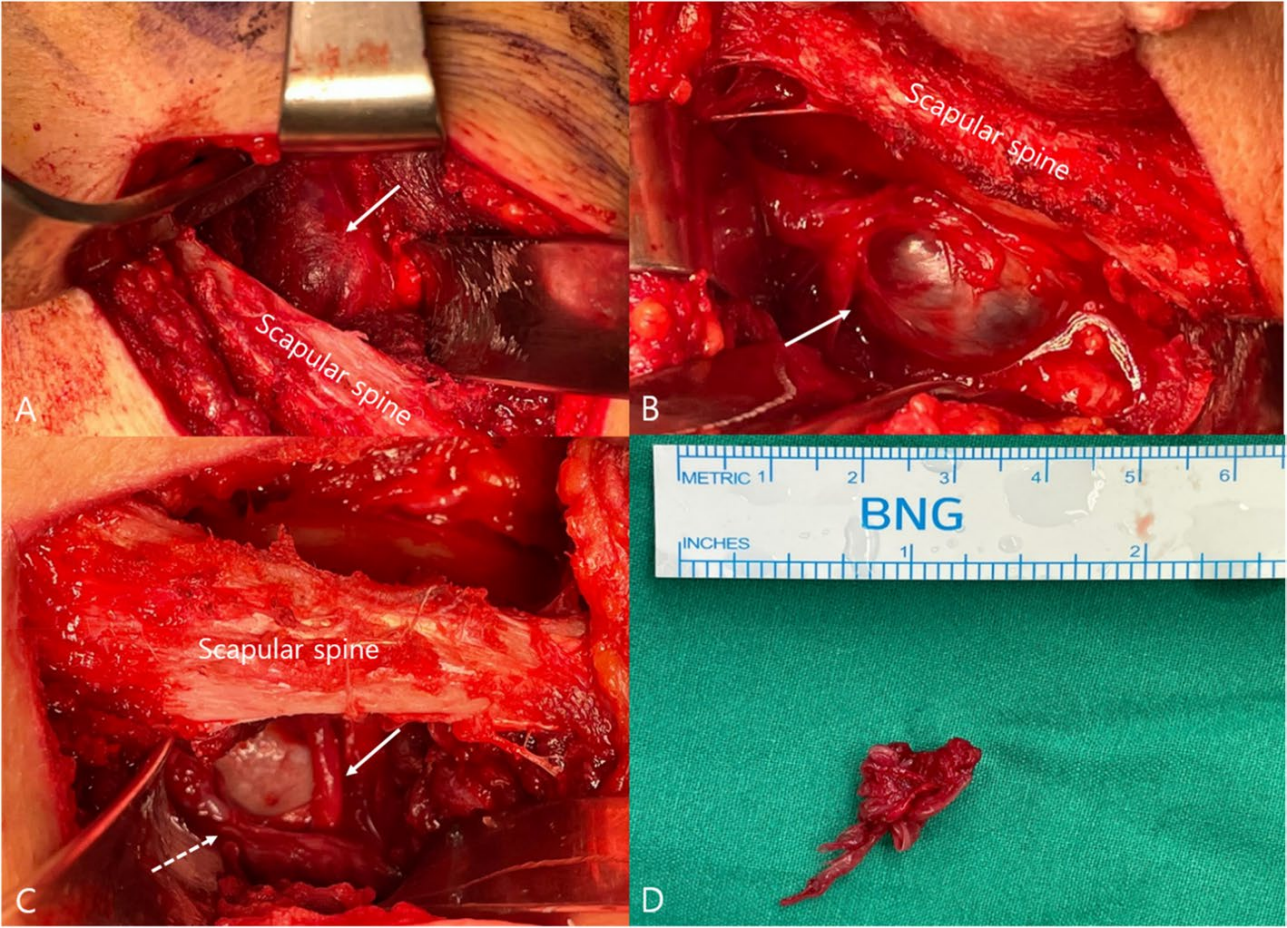
Intraoperative gross photo showing the cyst at the suprascapular notch. **A** The cyst (arrow) was positioned above the scapular spine. **B** The cyst (arrow) was widely enlarged under the scapular spine. **C** After cyst decompression, we were able to locate the suprascapular nerve (dotted arrow) and suprascapular artery (line arrow). **D** Intraoperative gross photo of the excised cyst

**Fig. 6:**
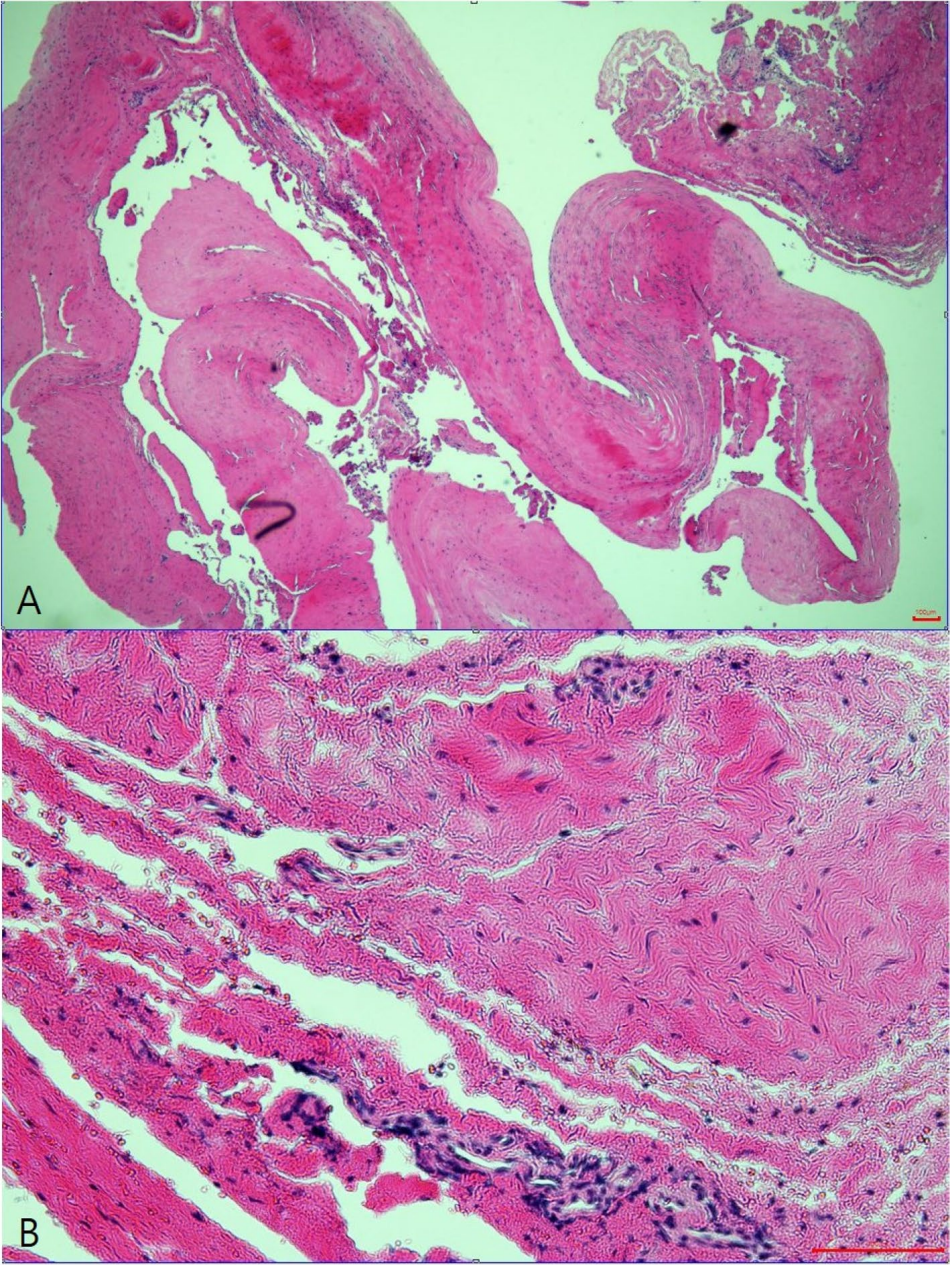
Hematoxylin and eosin-stained sections revealing fibrous tissue with hemosiderin-laden macrophages. **A** (× 40), **B** (× 200)

First, following minimal detachment of the deltoid muscle and retraction of the infraspinatus muscle, the spinoglenoid notch was exposed. Around the notch, a large, well-lobulated cyst was found compressing the suprascapular nerve (Fig. 5); hence, we gently isolated the cyst from the nerve and other soft tissues. However, we found that the cyst had spread beyond the scapular spine, near the suprascapular notch. We retracted the supraspinatus muscle and identified the cyst’s upper margin. To avoid iatrogenic injury, we carefully removed the entire cyst and performed decompression while directly visualizing the nerve. Finally, a biopsy was performed to achieve an accurate diagnosis. The biopsy results indicated “fibrous tissue with hemosiderin-laden macrophages,” suggesting that the cyst was a hematoma-like lesion (Fig. 6).

An arm sling was used for postoperative immobilization until the pain subsided (2 weeks). The patient performed gentle passive forward flexion exercises the next day after the surgery. Two weeks after the procedure, the arm sling was removed and active full range of motion exercise was recommended. At 6 months after the operation, the patient's muscle atrophy was slightly recovered, and there were no specific symptoms.

## Discussion

A rare cause of shoulder dysfunction is suprascapular nerve entrapment [9–11]. It could be caused on by a mass lesion, such as a paralabral cyst; trauma; repetitive overhead activities; or a massive rotator cuff tear as a result of traction injury [12–16]. Several authors have described various clinical presentations (e.g., shoulder pain, weakness, infraspinatus muscle atrophy, and posterior shoulder tenderness) in these patients [4]. Nonetheless, the primary complaint among their patients was pain, typically located in the posterior and lateral aspects of the shoulder, radiating down the arm. The pain was characterized as deep, dull, and diffuse [9]. In patients with severe neuropathy, atrophy and weakness of the supraspinatus and infraspinatus muscles may exist. In our case, the patient predominantly complained of dull and diffuse pain in the posterior shoulder. While infraspinatus muscle atrophy was visually evident, no muscle weakness was observed, and this phenomenon was confirmed by the normal EMG-NCV results. Post and Grinblat reported that EMG results may be normal in the very early stages of entrapment. They suggested that if the clinical features indicate the diagnosis of entrapment by a mass lesion (e.g., ganglion cyst or lipoma), and other conditions that potentially mimic nerve entrapment are excluded, surgical treatment may be indicated, despite normal EMG findings [9, 16].

As arthroscopic surgery has become more common in practice, several orthopedic surgeons currently treat suprascapular nerve entrapment using arthroscopy. One of its advantages is that it allows surgeons to treat nerve entrapment associated with labral, biceps, or cuff pathology concurrently [9]. However, arthroscopic decompression is not always applicable in cases of large cysts; thus, some surgeons still prefer open surgical decompression over the arthroscopic approach [4, 7, 17].

Open surgical decompression allows direct visualization and complete excision of the cyst while preventing its recurrence in cases involving a normal shoulder labrum [4]. As in our case, the patient presented with a large cyst in the normal shoulder labrum, and the cyst was suspected to be a hematoma, thus leading us to opt for the open excision technique. The suprascapular nerve normally lies close (18–23 mm) to the glenohumeral joint at the spinoglenoid notch level [18]. Therefore, surgeons should be cautious during open surgical decompression to prevent iatrogenic nerve injury.

As mentioned earlier, we used two approaches involving one incision. Due to the large cyst size, we proceeded to both the caudal and cephalad aspects of the scapular spine. Based on biopsy results, the cyst was confirmed to be a hematoma-like lesion. The patient stated that the pain in their shoulder usually occurred while overstraining their shoulder to “make a serve” while playing tennis. Presumably, the hematoma was filled with damaged muscle at that time. Previous studies have reported cases of ganglion or intraosseous cysts; however, to date, no cases involving nerve-compressing “hematoma” cysts have been reported, as demonstrated in our case. Based on the current case, even a hematoma resulting from excessive exercise can arguably cause nerve compression.

Our patient experienced significant posterior shoulder pain and noticeable infraspinatus atrophy on physical examination. Using MRI, the margins of a large cyst at the corresponding site were defined, whereas the labrum and rotator cuff were marked as normal. The primary treatment goal was pain resolution, which was fully and satisfactorily accomplished by open cyst decompression.

## Conclusion

Suprascapular nerve entrapment is a rare cause of posterior shoulder pain and dysfunction. A large spinoglenoid cyst resembling a hematoma, with a normal rotator cuff and glenoid labrum, has not been reported. We performed open surgical decompression of the suprascapular nerve and excised the cyst. Posterior shoulder pain promptly resolved without complications. This case report supports the consideration of suprascapular nerve entrapment in the differential diagnosis of posterior shoulder pain and highlights the importance of undressing the patient and examining the posterior shoulder for atrophy.

## Data Availability

The authors declare that data supporting the fndings of this study are available within the article.

## Abbreviations

SLAP: Superior labrum from anterior to posterior
MRI: Magnetic resonance imaging
EMG: Electromyography
NCV: Nerve conduction velocity
